# Cup of Tea

**Published:** 1857-12

**Authors:** 


### A CUP OF TEA

Is considered by many to be one of life's indispensabilities. To get the best cup out of the smallest amount of tea is worth knowing. Fill the teapot with boiling water, put in the tea, and let the pot stand five minutes; the leaves gradually sink, are not scalded, and the true aroma is retained, not lost, as is the case in the old-fashioned "tea-drawing."

